# Antidiabetic Properties, Bioactive Constituents, and Other Therapeutic Effects of* Scoparia dulcis*

**DOI:** 10.1155/2016/8243215

**Published:** 2016-08-10

**Authors:** Geethi Pamunuwa, D. Nedra Karunaratne, Viduranga Y. Waisundara

**Affiliations:** ^1^Department of Horticulture and Landscape Gardening, Faculty of Agriculture and Plantation Management, Wayamba University of Sri Lanka, Makandura, Gonawila, Sri Lanka; ^2^Department of Chemistry, Faculty of Science, University of Peradeniya, Peradeniya, Sri Lanka; ^3^Functional Food Product Development Project, National Institute of Fundamental Studies, Hantana Road, Kandy, Sri Lanka

## Abstract

This review discusses the antidiabetic activities of* Scoparia dulcis* as well as its antioxidant and anti-inflammatory properties in relation to the diabetes and its complications. Ethnomedical applications of the herb have been identified as treatment for jaundice, stomach problems, skin disease, fever, and kidney stones, reproductory issues, and piles. Evidence has been demonstrated through scientific studies as to the antidiabetic effects of crude extracts of* S. dulcis* as well as its bioactive constituents. The primary mechanisms of action of antidiabetic activity of the plant and its bioactive constituents are through *α*-glucosidase inhibition, curbing of PPAR-*γ* and increased secretion of insulin. Scoparic acid A, scoparic acid D, scutellarein, apigenin, luteolin, coixol, and glutinol are some of the compounds which have been identified as responsible for these mechanisms of action.* S. dulcis* has also been shown to exhibit analgesic, antimalarial, hepatoprotective, sedative, hypnotic, antiulcer, antisickling, and antimicrobial activities. Given this evidence, it may be concluded that* S. dulcis* could be promoted among the masses as an alternative and complementary therapy for diabetes, provided further scientific studies on the toxicological and pharmacological aspects are carried out through either* in vivo* or clinical means.

## 1. Introduction

A recent study published by the World Health Organization (WHO) in the Lancet as part of the NCD Risk Factor Collaboration (NCD-RisC) shows that the number of adults with diabetes has quadrupled worldwide in under four decades to 422 million, and the condition is fast becoming a major problem in poorer countries [1]. In one of the largest studies to date of diabetes trends, the researchers of NCD-RisC said aging populations and rising levels of obesity across the world mean diabetes is becoming a defining issue for global public health. This study used data from 4.4 million adults in different world regions to estimate age-adjusted diabetes prevalence for 200 countries. It found that between 1980 and 2014 diabetes has become more common among men than women, and rates of diabetes rose significantly in many low- and middle-income countries, including China, India, Indonesia, Pakistan, Egypt, and Mexico. While the alarming nature of the disease is as such, the inefficacy and inadequacy of current antidiabetic treatments in mitigating the disease condition have also been highlighted from studies such as the Linköping Diabetes Complications Study [2], the Diabetes Control and Complications Trial (DCCT) Research Group [3–5], and the UK Prospective Diabetes Study (UKPDS) Group [6–8]. Current treatments for diabetes include insulin therapy (for type 1 and some type 2 diabetes patients) and administration of oral hypoglycemic agents (i.e., insulin secretagogues, biguanides, *α*-glucosidase inhibitors, and insulin sensitizers), which nevertheless have been demonstrated to show deteriorations in hyperglycemic control and increased risks of contracting diabetic complications during long-term usage [9]. To further complicate the situation, the hefty expense of purchasing these drugs is a significant economic burden borne by the diabetic patients, especially given the increased prevalence of the disease in developing countries.

When taking historical elements into account from the perspective of antidiabetic treatments, diabetes is an ancient disease for which remedies have been detailed in many traditional medicinal pharmacopoeias. In Traditional Chinese Medicine (TCM), for instance, the disease has been referred to as “Xiaokezheng” or “Xiaodanzheng,” both of which mean diabetes [10]. In the Sri Lankan traditional medicinal system, the disease has been stated as “Madhumeha” [11]. In both of these traditional medicinal systems and many others, the usage of herbs for combating the disease condition and alleviating its symptoms has been practiced for many eons. Plants have always been an exemplary source of drugs and have directly or indirectly yielded many important medicines in the past. For instance, the discovery of the widely used hypoglycemic drug, Metformin, came from the traditional approach of using* Galega officinalis* [12]. Overall, herbal remedies are gaining popularity because of several advantages such as a comparatively lower incidence of side-effects at recommended dosages, better patient tolerance, relatively low cost, and acceptance due to a long history of use. From the perspective of diabetes, the more important cause is that herbal medicines provide rational means for remedying the disease condition as well as many other ailments which are obstinate and incurable in more Western systems of medicine. Therefore, a revival of interest in the use of plants in pharmacy has emerged worldwide recently from both the pharmaceutical industry as a source of new lead molecules and the general public who tend to use plant extracts in many ways as conventional and complementary therapies. Thus, bringing effective herbal remedies into the limelight especially those which have proven antidiabetic effects through at least* in vivo* and* in vitro* studies is of importance given the disturbing incidence of the disease, the voids of effective therapeutic remedies, and the tendency towards searching and promoting complementary and alternative therapies to combat and contain the progression of the disease.

**Figure 1:**
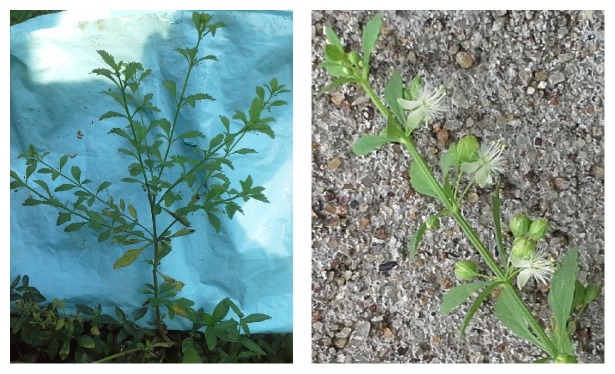
Leaf and flower structure of* S. dulcis* in its natural habitat.

**Figure 2:**
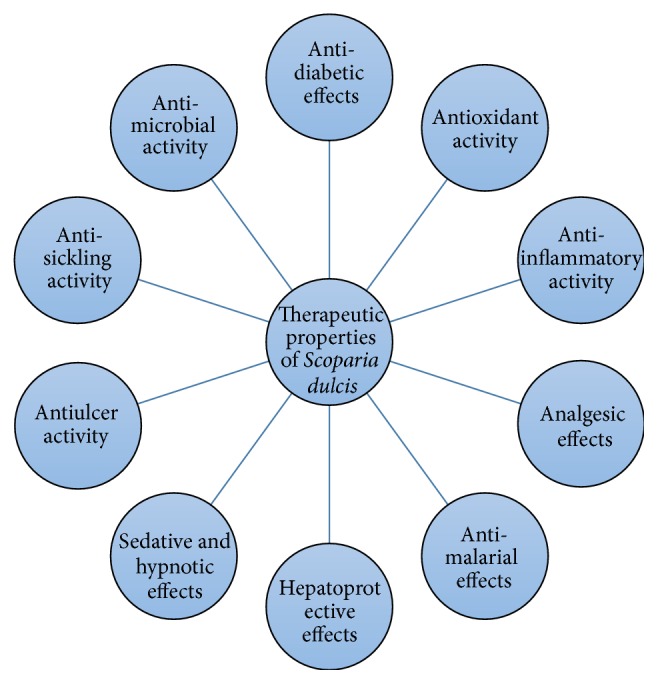
Schematic diagram displaying all the therapeutic properties of* S. dulcis*.

*Scoparia dulcis*, also known as sweet broomweed (family: Scrophulariaceae), is a perennial herb which is commonly found in tropical and subtropical regions.  Figure 1 shows the plant and its flowers in its natural habitat. The leaves of the plant are serrated and the flowers are white in colour.* S. dulcis* has been used in many traditional medicinal systems as an antidiabetic herb and for a variety of ailments. The plant is abundantly found in many countries and can be easily grown and cultivated, should there be a need for mass production. This review highlights the ethnomedicine, antidiabetic properties, antioxidant effects, bioactive chemical constituents, and other therapeutic properties of* S. dulcis*. A schematic outline showing all therapeutic properties of this plant is shown in Figure 2. Through this review, it is hoped that* S. dulcis* could be promoted for downstream scientific investigations where the herb as well as its bioactive constituents would be taken up for further clinical evaluations and thereby discover the true potential as a remedy for combating a global pandemic as well as other noncommunicable disease conditions at large.

## 2. Ethnomedicine

Before delving into the antidiabetic effects and other related properties of* S. dulcis* from a more scientific perspective, it is of importance to briefly summarize the ethnomedical applications of the herb, so that its therapeutic value is validated by its historical usage. The use of* S. dulcis* plant parts has been recorded from many parts of the world. In a very comprehensive study on the ethnomedical value of plants used in the preparation of traditional rice beer, Bhuyan and Baishya have identified the different plant parts used by several tribal groups in the State of Assam in India [13]. In this study, it was identified that Bodo, Karbi, Ahom, Deori, Rabha, Mising, and Sonowal Kachari tribes of Assam used widely differing herbs and plant parts in their rice beer preparations. Of these, the leaves of* S. dulcis* were used in particular by the Deori and Rabha tribes for alleviation of diabetes, jaundice, stomach problems, skin disease, and piles [13]. Bieski et al. conducted an ethnobotanical survey in the Nossa Senhora Aparecida do Chumbo District (NSACD), located in Poconé, Mato Grosso, Brazil and found that* S. dulcis* was used for several disease categories with diabetes being one of them [14]. In the South Indian State of Tamil Nadu, a survey of the phytotherapeutic agents used by the Nadars revealed that* S. dulcis* juice was taken orally to treat fever and kidney stones [15]. In Trinidad and Tobago,* S. dulcis* juice has been used for cooling babies and for reproductive problems in both men and women [16, 17], while, in Central Laos, in Bolikhamsai Province, the whole plant was used for treating nephritis [18].

Due to the prevalence of malaria in the Amazon Region, Ruiz et al. evaluated the antimalarial potential of traditional remedies used by the indigenous and Mestizo populations around the banks of the Nanay River, a tributary of the Amazon River in Loreto, Peru [19]. These researchers went as far as testing some of these plant extracts for activity on* Plasmodium falciparum*. They found that several of the plants used traditionally, including* S. dulcis*, had antiplasmodium activity. The whole plant of* S. dulcis* is recorded as one of many being used for the treatment of snakebite in the province of Antioquia in Colombia [20]. Over 90% of the bites in this province were caused by the snake* B. asper* (Viperidae) where around 4000 bites are reported annually in Columbia. The rapid effect of the toxins in the venom results in high mortality (5%) and serious long-term effects (6%). The healers and shamans administer plant extracts to neutralize the toxins such as phospholipase A2 present in the venom, where* S. dulcis* was identified as one of the plants being used for this purpose [21]. In Sri Lanka, rice based porridges containing herbal extracts and coconut milk have been traditionally consumed for breakfast. Senadheera et al. [22] reported that three porridges individually made with* Asparagus racemosus*,* Hemidesmus indicus,* or* Scoparia dulcis* were found to have a glycemic index value of less than 55, and peak blood glucose reduction values of around 40%. When diabetic Wistar rats were administered with these porridges, the porridge containing* S. dulcis* resulted in a reduced weight loss while exhibiting hypoglycemic and hypolipidaemic properties with no observed toxic effects.

## 3. Antidiabetic Properties

### 3.1. Evidence from Studies Based on Evaluating the Antidiabetic Properties of Whole Extracts of* S. dulcis*

**Figure 3:**
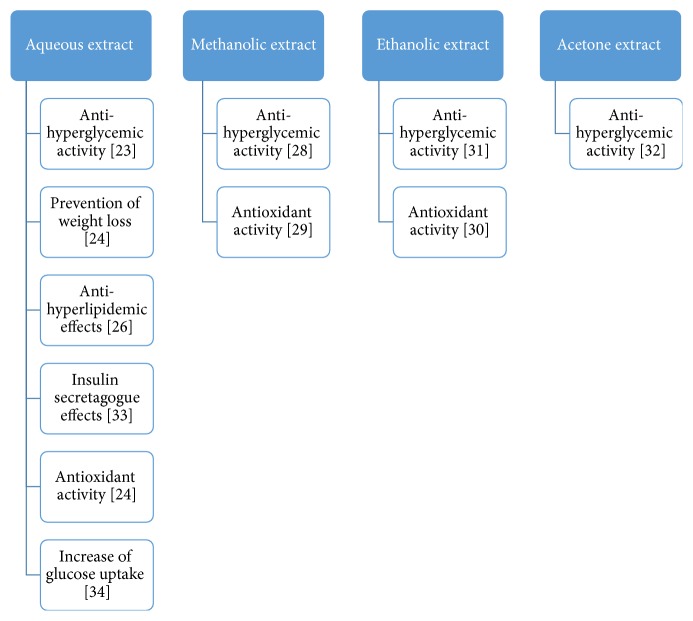
Diagrammatic representation depicting the various actions of different fractions of* S. dulcis*.

Extracts of* S. dulcis* plant as a whole and numerous compounds isolated from* S. dulcis* all have exhibited antidiabetic or hypoglycemic properties. For this section of the review, the antidiabetic property of* S. dulcis* which was demonstrated by studies based on various types of extracts of the whole plant is highlighted briefly. A diagrammatic representation representing the various actions of different solvent fractions is shown in Figure 3.

Pari and Venkateswaran demonstrated hypoglycemic activity of the aqueous extract of* S. dulcis* leaves using alloxan induced hyperglycemic rats [23]. They, further, indicated that this hypoglycemic effect was highly pronounced when 0.45 g/kg (body weight) of the extract was administrated orally for 45 days. In addition to hypoglycemic effect, the administration of this plant extract prevented weight loss which is characteristic of diabetic patients [24]. Furthermore, it has been shown that the aqueous extract of* S. dulcis*, when administered at a dose of 200 mg/kg (body weight), exhibits a hypoglycemic effect on streptozotocin induced diabetic rats [24]. Similarly, Das and Chakraborty [25] and Attanayake et al. [26] used streptozotocin induced diabetic rats to demonstrate the antihyperglycemic effect of the aqueous extracts of* S. dulcis*. In addition to possessing antidiabetic effects, the aqueous extracts of* S. dulcis* exhibit antihyperlipidemic effects [26]. For example, streptozotocin induced diabetic rats have shown decreased levels of lipids including cholesterol, triglycerides, fatty acids, and phospholipids, decreased levels of very low density lipoprotein and low density lipoprotein cholesterol, and reduced 3-hydroxy-3-methylglutaryl (HMG) CoA reductase activity, as a result of oral administration of the aqueous extract of* S. dulcis* (200 mg/kg of body weight) for 6 weeks [27].

Apart from aqueous extracts, numerous studies have shown that polar organic extracts such as methanol and ethanol extracts of* S. dulcis* possess antidiabetic properties. For example, Sharma and Shah illustrated using streptozotocin induced diabetic rats [28] that the flavonoids from the methanol extract of the aerial parts of* S. dulcis* impart an antihyperglycemic activity comparable to Glibenclamide. Strengthening their claim, Mishra et al. reported that both the aqueous and methanol extracts of* S. dulcis* contain polyphenols that these extracts show antioxidant activity which may most probably be due to the polyphenols and that there is a correlation between the antidiabetic activity and antioxidant activity of these extracts [29]. Another study conducted by Zulfiker et al. using alloxan induced diabetic mice revealed that the ethanol extract of the aerial parts of the plant shows antidiabetic activity [30]. Further, this study also exhibited that this extract shows moderate antioxidant activity compared to ascorbic acid, using DPPH (1,1-diphenyl-2-picrylhydrazyl) assay and phosphomolybdenum assay. In addition to the extracts of the whole plant and aerial parts of the plant, the root of* S. dulcis* exhibits significant antidiabetic activity according to Reddy et al. [31]. Specifically, the hydroalcoholic extract of the roots of this plant had imparted a hypoglycemic activity similar to that of Glibenclamide, on alloxan induced rats in this particular study. Interestingly, a comparison of hypoglycemic activity of* S. dulcis* extracts in different organic solvents was carried out, using streptozotocin induced diabetic rats, by Talukder et al. who revealed that the acetone extract was most effective followed by methanol, petroleum ether, and ethanol extracts [32].

The mechanisms of action of* S. dulcis* plant extracts possessing antidiabetic activity have also been elucidated. It has been demonstrated that the antidiabetic activity of the aqueous extracts of* S. dulcis* may be attributable to its insulin secretagogue activity. For example, pancreatic islets isolated from mice have shown a 6-fold increase in secretion of insulin when the islets were exposed to an aqueous extract of* S. dulcis* at a dose of 10 *μ*g/mL [33]. Also,* S. dulcis* imparts its antidiabetic effects via altering the levels of many antioxidant enzymes and enzymes of the polyol pathway. In fact, Latha and Pari showed, using streptozotocin induced diabetic rats, that the aqueous extract of* S. dulcis* significantly decreased the level of sorbitol dehydrogenase while increasing the levels of the antioxidant enzymes glutathione peroxidase and glutathione-S-transferase [24]. Beh et al. demonstrated using L6 rat myoblasts (CRL-1458) that the TLC fraction 7 of the aqueous extract of* S. dulcis* possesses glucose uptake activity comparable to that of insulin [34]. Moreover, they revealed that the TLC fraction 7 of the aqueous extract of* S. dulcis* is more capable than insulin of glucose transport that may be partly responsible for its antidiabetic activity.

### 3.2. Evidence from Studies Based on Evaluating the Antidiabetic Properties of Bioactive Constituents of* S. dulcis*

**Figure 4:**
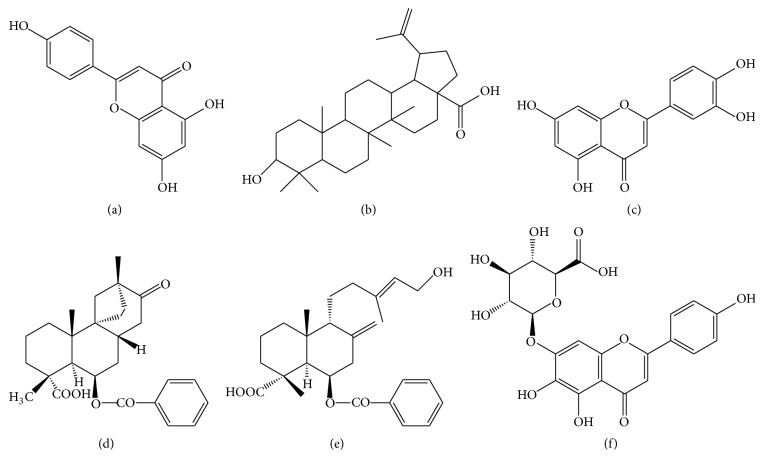
Chemical constituents in* S. dulcis* which have demonstrated PPAR-*γ* and *α*-glucosidase inhibitory activities: (a) apigenin, (b) betulinic acid, (c) luteolin, (d) scopadulcic acid B, (e) scoparic acid A, and (f) scutellarin.

**Figure 5:**
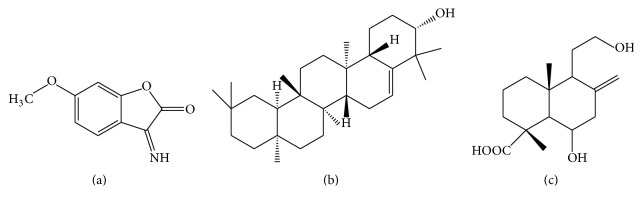
Chemical constituents in* S. dulcis* which have demonstrated insulin secretagogue activities: (a) coxicol, (b) glutinol, and (c) scoparic acid D.

The antidiabetic properties of* S. dulcis* have been primarily owed to the existence of diterpenes [35–38], triterpenes [39, 40], and flavonoids [41] which have been mostly isolated from the aerial parts of the plant. A variety of chemical constituents have led to a plethora of antidiabetic pathways of action initiated by the plant, such as *α*-glucosidase and peroxisome proliferator-activated receptor gamma (PPAR-*γ*) agonistic and insulin secretagogue activities. Some of the compounds which have displayed these antidiabetic mechanisms of action by way of* in vitro* and* in vivo* studies are shown in Figures 4 and 5. These pathways of action and the respective compounds responsible for these properties with supporting evidence as provided through* in vitro* and* in vivo* studies are briefly discussed herein.

#### 3.2.1. *α*-Glucosidase Inhibitory Activities

The use of enzyme inhibitors produces carbohydrate malabsorption and, hence, moderates blood glucose and insulin elevation [42]. This characteristic in particular is shown to be beneficial in the treatment of type 2 diabetes patients [43]. The digestive process of starch involves salivary *α*-amylase, pancreatic *α*-amylase, and the small intestinal brush border *α*-glucosidase, that is, maltase-amyloglucosidase and sucrase-isomaltase [44–46]. While inhibition of all 3 of these enzymes is deemed important in terms of control and release of blood glucose levels, inhibition of *α*-glucosidase in particular is considered as a priority, given that it is the final step upon which the rate of breakdown of carbohydrates is decided. Acarbose is one of the commonly used synthetic starch hydrolase inhibitors which is prescribed as an antidiabetic drug. However, it has been known to cause adverse health effects such as promotion of hypoglycemia at higher doses, as well as diarrhea and flatulence due to the gut microflora fermentation on the undigested carbohydrates [47]. Thus, the occurrence of natural starch hydrolase inhibitors is currently being explored, an aspect in which compounds of* S. dulcis* have been investigated as well. In the study by Liu et al. [48], the* in vitroα*-glucosidase inhibitory activities of the flavonoids scutellarein, apigenin, and luteolin and the terpenoids scopadulcic acid B and betulinic acid which were present in* S. dulcis* were observed to be more potent than the positive control, acarbose (with IC_50_ values in the range 13.7−132.5 *μ*M). A confirmation of these properties of the bioactive compounds was stated by Mishra et al. in their review [49].

#### 3.2.2. PPAR-*γ* Agonistic Activities

PPAR-*γ* is a member of the nuclear hormone receptor superfamily which can be activated by many kinds of ligands, such as flavonoids, terpenoids, and unsaturated fatty acids [50–52]. Its activation results in increased insulin sensitivity partly by reversing lipotoxicity-induced insulin resistance [53]. Thiazolidinediones (TZDs), which are well-known insulin resistance ameliorating agents in use for the treatment of type 2 diabetes since the 1980s, have been identified as potent PPAR-*γ* inhibitors [48]. Nevertheless, as with many of the synthetic drugs in use for diabetes, TZDs are also fraught with many side-effects [48]. Thus, screening of new PPAR-*γ* agonists from herbal medicine seems a reasonable strategy for the discovery of antidiabetic agents or their lead compounds. In the study by Liu et al. [48], scoparic acid A, scutellarein, apigenin, and luteolin had exhibited PPAR-*γ* agonistic activities* in vitro*, with EC_50_ values ranging from 0.9 to 24.9 *μ*M. This study could be considered as the first of its kind to report the PPAR-*γ* agonistic activities of apigenin and luteolin in particular. Through this study, it was also evident that the bioactive constituents impart antidiabetic properties through multiple biochemical pathways, with individual compounds being able to modulate and mitigate the disease condition through numerous mechanisms of action.

#### 3.2.3. Insulin Secretagogues

The importance of insulin secretagogues as a remedy for combating diabetes was highlighted in the Kyoto declaration of 2013, where nonobese type 2 diabetic patients mostly of Asian origin were observed to possess defects in insulin secretion rather than insulin resistance [54]. The insulin secretory activity and cytoprotective properties of aqueous* S. dulcis* extract have been previously reported by Latha et al. [55]. Identification of compounds responsible for these properties was also reported by Sharma et al. [56]. In this study, coixol and glutinol (Figure 4) were found to be potent and mildly active, respectively, in terms of insulin secretagogue activity. Coixol was further evaluated for insulin secretory activity on MIN-6 cells and was further subjected to* in vitro* cytotoxicity assay against MIN-6, 3T3 cell lines, and islet cells and* in vivo* acute toxicity test in mice which was found to be nontoxic. Thus, the study confirmed the insulin secretory activity of coixol and glutinol which supported the ethnobotanic uses of* S. dulcis* as an antidiabetic agent containing potent insulin secretagogues. Additionally, the study by Latha et al. [55] further verified the insulin secretagogue properties of scoparic acid D (SAD) in particular. In brief, streptozotocin (STZ) induced diabetic Wistar rats were administered with SAD at dosages of 10, 20, and 40 mg/kg bodyweight for 15 days. At the end of the experimental period, the SAD-treated STZ diabetic rats showed decreased levels of glucose as compared with diabetic control rats. The improvement in blood glucose levels of SAD-treated rats was associated with a significant increase in plasma insulin levels. SAD at a dose of 20 mg/kg bodyweight exhibited a significant effect when compared with other doses. Further, the effect of SAD was tested on STZ-treated rat insulinoma cell lines (RINm5F cells) and isolated islets* in vitro*. SAD at a dose of 20 mg/mL had evoked twofold stimulation of insulin secretion from isolated islets, indicating its insulin secretagogue activity. This study also demonstrated the cytoprotective effects of SAD.

## 4. Other Therapeutic Properties of* S. dulcis* Relevant to Diabetes

Oxidative stress and elevated inflammatory levels have been biochemically determined to be the root cause of diabetic complications as shown by epidemiological and biochemical data [57, 58]. Thus, the antioxidant and anti-inflammatory properties of* S. dulcis* are discussed here, in view of the potential of this plant to mitigate the deleterious effects resulting from hyperglycemia-induced oxidative stress and inflammatory reactions.

### 4.1. Antioxidant Activity of* S. dulcis*

Several researchers have studied the effect of* S. dulcis* extracts on the ability to scavenge free radicals. Though many report the activity of the water extract, some have reported the activity of methanolic, ethanolic, chloroform, and hexane extracts. Babincová and Sourivong used the DPPH assay to demonstrate strong antioxidant activity of the extract [59]. Coulibaly et al. [60] studied the antioxidant property of the hexane, chloroform, and methanol extracts of the plant by the DPPH and FRAP assays. In addition, inhibition of lipid peroxidation was measured by the TBARS assay and inhibition of lipoxygenase and xanthine oxidase by the extracts was determined [60]. They found that the chloroform extract exhibited the highest activity and concluded that the phytochemical content being the highest in this extract was the responsible factor for the observation [61, 62]. Three extracts, aqueous, ethanolic, and chloroform, were shown to have significant antioxidant capacity [DPPH, FRAP, *β*-carotene bleaching, and (TBARS) assay] [63, 64]. The water extract showed the highest activity. Zulfiker et al. studied the effect of the ethanolic extract of S.* dulcis* on alloxan induced diabetic mice [30]. The extract, at a dose of 100 and 200 mg/kg, reduced glucose level by 31.87% and 46.97%, respectively, while a 50.74% reduction was given by Metformin after 2 weeks. The antioxidant potential assessed by DPPH free radical scavenging assay was moderate in comparison to ascorbic acid (IC_50_ of 243.82 *μ*g/mL for plant extract and 58.92 *μ*g/mL for ascorbic acid).

### 4.2. Anti-Inflammatory Activity of* S. dulcis*

Inflammation is a complex nonspecific immune response triggered by damage to living tissues that protects higher organisms from infection and injury. There are two types of inflammation, and their effects can be either beneficial (defense against agents interfering with homeostasis, that is, acute inflammation) or harmful (causing damage to cells and tissues, that is, chronic inflammation) [65]. de Farias Freire et al. reported that the ethanol extracts were superior to water extracts of* S. dulcis* in eliciting effects probably related to the anti-inflammatory activity of the plant [66]. Ethnobotanical studies in many parts of the world have revealed the use of* S. dulcis* for anti-inflammatory activity [67, 68]. Since anti-inflammatory medications are used to relieve menstrual discomfort, plants having anti-inflammatory activity may explain their use in traditional medicines for relieving these symptoms. Hence, Michel et al. researched into plants used to treat symptoms related to menstruation and menopause by the Q'eqchi, the third largest Maya population in Guatemala [69]. They found that* S. dulcis* was used to alleviate labour pains. An ethnobotanical survey conducted by Souza et al. in north eastern Brazil indicated that root of* S. dulcis* was used for treating inflammation and uterine inflammation [70]. Sala et al. [71] have investigated the anti-inflammatory pathway of* S. dulcis* aqueous extracts* in silico* and have discovered its ability to inhibit human inhibitor nuclear-factor *κ*B kinase 2 (hIKK-2). hIKK-2 is a serine-threonine protein kinase belonging to the IKK complex and is the primary component responsible for activating nuclear-factor *κ*B transcription factor (NF-*κ*B) in response to inflammatory stimuli. The NF-*κ*B pathway is deemed important in the regulation of gene expression controlling cellular immune and inflammatory responses and has motivated research groups in both academia and the pharmaceutical industry to devote increasing efforts to developing synthetic ATP-competitive inhibitors for hIKK-2 [72]. Thus, the hIKK-2 inhibitory activity of* S. dulcis* could be considered as an important therapeutic characteristic in its promotion as an anti-inflammatory agent.

## 5. Other Therapeutic Properties of* S. dulcis*

Several systematic studies have been carried out on various other therapeutic properties of* S. dulcis*. While these studies are not numerous as compared with those on antidiabetic, antioxidant, and anti-inflammatory activities, the outcomes could be considered as stepping stones for investigating these therapeutic properties of* S. dulcis* in detail, through further* in vitro*,* in vivo,* or clinical means.

### 5.1. Analgesic Properties

The analgesic properties of the plant have been substantiated by* in vitro* investigations which have been verified to have extended from the existence of glutinol [30, 73]. Additionally, the analgesic and hyperanalgesic properties of* S. dulcis* aqueous extracts have been verified in rat models by Ratnasooriya et al. [74]. This is an important characteristic in possession by this plant since current analgesics, especially opiates, have been known to cause adverse side-effects such as gastric lesions.

### 5.2. Antimalarial Properties

The antimalarial effects of* S. dulcis* have been investigated by Bourdy et al. [75]. Aqueous extracts of the aerial part of* S. dulcis* were explored for the inhibitory activity versus* Plasmodium falciparum*. Although the antimalarial effects were not as significant in comparison with the rest of the plants selected for this particular study, positive observations were noted in terms of its activity against the parasite.

### 5.3. Hepatoprotective Effects

Hepatoprotective effects of* S. dulcis* have been observed by Li et al. [10] in mice containing CCl_4_-induced acute liver injuries. In this study, an oral dose of 800 mg/kg had exhibited a significant (*P* < 0.01) protective effect against the CCl_4_-induced changes in serum aspartate aminotransferase (ASAT), alanine aminotransferase (ALAT), alkaline phosphatase (ALP), total protein (TP), and liver histopathology, compared with the positive control. Similar observations have been made by Ediriweera and Ratnasooriya [11], where crude ethanolic and aqueous extracts of* S. dulcis* were evaluated against CCl_4_-induced liver cirrhosis in Sprague Dawley rats. These values had been comparable with the standard, and silymarin and were associated with the ability of* S. dulcis* to scavenge free radicals. Both of these studies provided evidence as to the traditional practice of administering* S. dulcis* for hepatic ailments.

### 5.4. Sedative and Hypnotic Effects

The study by Moniruzzaman et al. [76] evaluated the sedative and hypnotic effect of the ethanolic extract of whole plants of* S. dulcis*. The sedative and hypnotic activity were then investigated using hole cross, open field, hole-board, rotarod, and thiopental sodium-induced sleeping time determination tests in mice at the doses of 50, 100, and 200 mg/kg of the ethanolic extracts of the plant. A significant dose-dependent inhibition of locomotor activity of mice in both hole cross and open field tests was observed in this instance, suggesting that* S. dulcis* may possess sedative principles with potent hypnotic properties.

### 5.5. Antiulcer Activity

Among other miscellaneous therapeutic properties, the study by Babincová et al. [77] explored the antiulcer activity of water extracts of* S. dulcis* in Sprague-Dawley rats. For the first time,* S. dulcis* water extract was verified to possess gastroprotective activity as evidenced by its significant inhibition in the formation of ulcers induced by indomethacin in this study.

### 5.6. Antisickling Activity

The antisickling activity of* S. dulcis* was recently investigated by Abere et al. [78]. Sickle cell disease (SCD) is one of the most prevalent morbidity and mortality diseases in Africa [79]. Management of SCD is aimed at relieving pain, preventing infections and management of complications. First-line clinical management of sickle cell anaemia includes use of hydroxyurea, folic acid, amino acids, and blood transfusion to stabilize the patient's haemoglobin level [80]. These are quite expensive and have attendant risk factors, thereby gradually paving way for the consideration of condiments from natural sources as antisickling remedies [81]. Ethnomedicinally,* S. dulcis* is used to manage sickle cell disease in Nigeria [78]. Abere et al. [78] confirmed traditional usage of* S. dulcis* in the management of SCD and a candidate for further investigations.

### 5.7. Antimicrobial Activity

In the study by Coulibaly et al. [82], the antimicrobial activity of the acetone : water (70 : 30) extract of* S. dulcis* was investigated against the bacterial cultures of* Bacillus licheniformis*,* Escherichia coli*,* Klebsiella pneumonia*,* Pseudomonas aeruginosa,* and* Staphylococcus aureus *and the fungal cultures of* Gloeophyllum trabeum*,* Pycnoporus sanguineus*,* Fomitopsis palustris*,* Schizophyllum commune,* and* Trametes versicolor*. They concluded that the antifungal activity of* S. dulcis* was more pronounced than its antibacterial activity in this study. Niveditha and Prabavathy [83] tested for the ability of the ethanolic extract of the leaves of the plant to inhibit virulence factors of the two pathogens* Escherichia coli* and* Staphylococcus aureus*. They used untreated and ethanolic extract treated bacteria to study the effect of the extract on inhibition of haemagglutination, haemolysis, proteolysis, lipolysis, and gelatinase production. The ability of the extract to inhibit the tests indicates the potential of* S. dulcis* for use as a commercial drug against urinary tract infections. Uma et al. had tested six extracts (petroleum ether, toluene, chloroform, methanol, ethanol, and water) of* S. dulcis* for activity towards the gram positive strains* Bacillus* sp. and* Corynebacterium *sp. and the gram negative bacteria* Escherichia coli*,* Klebsiella pneumoniae,* and* Acinetobacter* sp. using the agar well diffusion assay [84]. They report activity of the extracts towards both gram positive and gram negative strains. However the activity of the extracts was mild in comparison to the ampicillin standard which was used. In another study on the ethanolic extract of S.* dulcis*, Zulfiker et al. performed disc diffusion assays on several gram positive and negative strains [85]. Their results also indicated very mild activity of the extract compared to the kanamycin standard. When they tested the same extract against three fungal strains (*Aspergillus niger*,* Saccharomyces cerevisiae,* and* Candida albicans*), the results indicated smaller zones of inhibition (6–10 mm) in comparison to that of the nystatin standard (20–25 mm).

## 6. Conclusions

It is evident from the scientific studies carried out to date on* S. dulcis* that this plant is worthy of being explored and promoted as complementary and alternative means of combating diabetes. Additional biochemical avenues of exploration in this aspect would be to evaluate whether the extracts or bioactive compounds are capable of decreasing insulin resistance which is a typical characteristic of type 2 diabetes. Studies which go in proximity to exploring this characteristic have been carried out but none which specifically focuses on type 2 diabetes and insulin resistance. This could be potentially explored using genetically modified rat models such as the Goto-Kakizaki model. Additionally, it would be of value to investigate whether the plant and its bioactive compounds are capable of regenerating pancreatic *β*-cells. This aspect could be easily and systematically verified using* in vitro* and* in vivo* studies. Nevertheless, as compared with other types of plants which have been ethnomedically used for diabetes, it is possible that* S. dulcis* has progressed quite notably in terms of scientific studies, from evaluation of crude extracts, to identification of chemical constituents, to identification of bioactive compounds and discovering their specific mechanisms of action. Additionally, although the plant may specifically be promoted for antidiabetic purposes, given the scientific evidence as to its miscellaneous therapeutic properties, it may also be used for overall health and wellness purposes. Nevertheless, despite the plethora of systematic studies on* S. dulcis*, it is imperative to investigate the toxicological and pharmacological aspects of the plant either through* in vivo* or clinical means. As with many other herbal remedies, while the plant is currently being administered or consumed by way of traditional practices, identification of recommended dosages and consumption limits should be determined before formal promotion of the plant, its extracts, or bioactive constituents for antidiabetic or other therapeutic purposes.

## References

[1] NCD Risk Factor Collaboration (NCD-RisC) (2016). Worldwide trends in diabetes since 1980: a pooled analysis of 751 population-based studies with 4·4 million participants. *The Lancet*.

[2] Nordwall M., Bojestig M., Arnqvist H. J., Ludvigsson J. (2004). Declining incidence of severe retinopathy and persisting decrease of nephropathy in an unselected population of Type 1 diabetes: the Linköping Diabetes Complications Study. *Diabetologia*.

[3] The Diabetes Control and Complications Trial (DCCT) Research Group (1993). The effect of intensive treatment of diabetes on the development and progression of long-term complications in insulin-dependent diabetes mellitus. *The New England Journal of Medicine*.

[4] The Diabetes Control and Complications Trial Research Group (DCTT) (1997). Clustering of long-term complications in families with diabetes in the diabetes control and complications trial. *Diabetes*.

[5] The Diabetes Control and Complications Trial (DCTT) (2000). Retinopathy and nephropathy in patients with type 1 diabetes four years after a trial of intensive therapy. *The New England Journal of Medicine*.

[6] UK Prospective Diabetes Study (UKPDS) (1998). Relative efficacy of sulfonylurea, insulin and metformin therapy in newly diagnosed non-insulin dependent diabetes with primary diet failure followed for six years (UKPDS 24). *Annals of Internal Medicine*.

[7] Turner R. (1998). Intensive blood-glucose control with sulphonylureas or insulin compared with conventional treatment and risk of complications in patients with type 2 diabetes (UKPDS 33). *The Lancet*.

[8] UK Prospective Diabetes Study Group (UKPDS) (1998). Association of hyperglycemia with macrovascular and microvascular complications of type 2 diabetes: a prospective observational study (UKPDS 35). *British Medical Journal*.

[9] Eckel R. H., Grundy S. M., Zimmet P. Z. (2005). The metabolic syndrome. *The Lancet*.

[10] Li W. L., Zheng H. C., Bukuru J., De Kimpe N. (2004). Natural medicines used in the traditional Chinese medical system for therapy of diabetes mellitus. *Journal of Ethnopharmacology*.

[11] Ediriweera E. R. H. S. S., Ratnasooriya W. D. (2009). Review on herbs used in the treatment of diabetes mellitus by Sri Lankan Ayurvedic and traditional physicians. *Ayu*.

[12] Das S. K., Chakrabarti R. (2005). Non-insulin dependent diabetes mellitus: present therapies and new drug targets. *Mini-Reviews in Medicinal Chemistry*.

[13] Bhuyan B., Baishya K. (2013). Ethno medicinal value of various plants used in the preparation of traditional rice beer by different tribes of Assam, India. *Drug Invention Today*.

[14] Bieski I. G. C., Rios Santos F., De Oliveira R. M. (2012). Ethnopharmacology of medicinal plants of the pantanal region (Mato Grosso, Brazil). *Evidence-Based Complementary and Alternative Medicine*.

[15] Jeeva S., Femila V. (2012). Ethnobotanical investigation of Nadars in Atoor village, Kanyakumari District, Tamilnadu, India. *Asian Pacific Journal of Tropical Biomedicine*.

[16] Lans C. A. (2006). Ethnomedicines used in Trinidad and Tobago for urinary problems and diabetes mellitus. *Journal of Ethnobiology and Ethnomedicine*.

[17] Lans C. (2007). Ethnomedicines used in Trinidad and Tobago for reproductive problems. *Journal of Ethnobiology and Ethnomedicine*.

[18] Libman A., Bouamanivong S., Southavong B., Sydara K., Soejarto D. D. (2006). Medicinal plants: an important asset to health care in a region of Central Laos. *Journal of Ethnopharmacology*.

[19] Ruiz L., Ruiz L., MacO M., Cobos M., Gutierrez-Choquevilca A.-L., Roumy V. (2011). Plants used by native Amazonian groups from the Nanay River (Peru) for the treatment of malaria. *Journal of Ethnopharmacology*.

[20] Kpodar M. S., Lawson-Evi P., Bakoma B. (2015). Ethnopharmacological survey of plants used in the treatment of diabetes mellitus in south of Togo (Maritime Region). *Journal of Herbal Medicine*.

[21] Vásquez J., Jiménez S. L., Gómez I. C. (2013). Snakebites and ethnobotany in the Eastern region of Antioquia, Colombia—the traditional use of plants. *Journal of Ethnopharmacology*.

[22] Senadheera S. P. A. A. S. U., Ekanayake S., Wanigatunge C. (2014). Anti-diabetic properties of rice-based herbal porridges in diabetic Wistar rats. *Phytotherapy Research*.

[23] Pari L., Venkateswaran S. (2002). Hypoglycaemic activity of *Scoparia dulcis* L. extract in Alloxan induced hyperglycaemic rats. *Phytotherapy Research*.

[24] Latha M., Pari L. (2004). Effect of an aqueous extract of *Scoparia dulcis* on blood glucose, plasma insulin and some polyol pathway enzymes in experimental rat diabetes. *Brazilian Journal of Medical and Biological Research*.

[25] Das H., Chakraborty U. (2011). Anti-hyperglycemic effect of *Scoparia dulcis* in streptozotocin induced diabetes. *Research Journal of Pharmaceutical, Biological and Chemical Sciences*.

[26] Attanayake A. P., Jayatilaka K. A. P. W., Pathirana C., Mudduwa L. K. B. (2015). Acute hypoglycemic and antihyperglycemic effects of ten Sri Lankan medicinal plant extracts in healthy and streptozotocin induced diabetic rats. *International Journal of Diabetes in Developing Countries*.

[27] Pari L., Latha M. (2006). Antihyperlipidemic effect of Scoparia dulcis (Sweet Broomweed) in streptozotocin diabetic rats. *Journal of Medicinal Food*.

[28] Sharma V. J., Shah U. D. (2010). Antihyperglycemic activity of flavonoids from methanolic extract of aerial parts of *Scoparia dulcis* in streptozotocin induced diabetic rats. *International Journal of ChemTech Research*.

[29] Mishra M. R., Mishra A., Pradhan D. K., Panda A. K., Behera R. K., Jha S. (2013). Antidiabetic and antioxidant activity of *Scoparia dulcis* Linn. *Indian Journal of Pharmaceutical Sciences*.

[30] Zulfiker A. H. Md., Ripa F. A., Rahman M. M. (2010). Anti-diabetic and antioxidant effect of *Scoparia dulcis* in alloxan induced albino mice. *International Journal of PharmTech Research*.

[31] Reddy S. K., Kumar S. A., Ganapaty S. (2012). Pharmacological screening of *Scoparia dulcis* roots for hypoglycaemic activity. *International Journal of Pharmacy and Pharmaceutical Sciences*.

[32] Talukder A., Choudhury M. D., De B. (2013). Hypoglycaemic activity of *Scoparia dulcis* L. in different solvent systems. *International Journal of Pharmacy and Pharmaceutical Sciences*.

[33] Latha M., Pari L., Sitasawad S., Bhonde R. (2004). Insulin-secretagogue activity and cytoprotective role of the traditional anti-diabetic plant *Scoparia dulcis* (Sweet Broomweed). *Life Sciences*.

[34] Beh J. E., Latip J., Abdullah M. P., Ismail A., Hamid M. (2010). *Scoparia dulcis* (SDF7) endowed with glucose uptake properties on L6 myotubes compared insulin. *Journal of Ethnopharmacology*.

[35] Hayashi T., Kawasaki M., Okamura K. (1992). Scoparic acid A, a *β*-Glucuronidase inhibitor from *Scoparia dulcis*. *Journal of Natural Products*.

[36] Hayashi T., Kishi M., Kawasaki M. (1987). Scopadulcic acid-A and -B, new diterpenoids with a novel skeleton, from a *Paraguayan crude* drug ‘typychá kuratũ’ (*Scoparia dulcis* L.). *Tetrahedron Letters*.

[37] Hayashi T., Okamura K., Tamada Y., Iida A., Fujita T., Morita N. (1993). A new chemotype of *Scoparia dulcis*. *Phytochemistry*.

[38] Ahmed M., Jakupovic J. (1990). Diterpenoids from *Scoparia dulcis*. *Phytochemistry*.

[39] Tsai J.-C., Peng W.-H., Chiu T.-H., Lai S.-C., Lee C.-Y. (2011). Anti-inflammatory effects of *Scoparia dulcis* L. and betulinic acid. *The American Journal of Chinese Medicine*.

[40] Mahato S. B., Das M. C., Sahu N. P. (1981). Triterpenoids of *Scoparia dulcis*. *Phytochemistry*.

[41] Kawasaki M., Hayashi T., Arisawa M., Morita N., Berganza L. H. (1988). 8-Hydroxytricetin 7-glucuronide, a *β*-glucuronidase inhibitor from *Scoparia dulcis*. *Phytochemistry*.

[42] Delorme S., Chiasson J.-L. (2005). Acarbose in the prevention of cardiovascular disease in subjects with impaired glucose tolerance and type 2 diabetes mellitus. *Current Opinion in Pharmacology*.

[43] van de Laar F. A., Lucassen P. L., Akkermans R. P., Van De Lisdonk E. H., Rutten G. E., Van Weel C. (2005). *α*-Glucosidase inhibitors for patients with type 2 diabetes: results from a Cochrane systematic review and meta-analysis. *Diabetes Care*.

[44] Van Beers E. H., Büller H. A., Grand R. J., Einerhand A. W. C., Dekker J. (1995). Intestinal brush border glycohydrolases: structure, function, and development. *Critical Reviews in Biochemistry and Molecular Biology*.

[45] Lebovitz H. E. (1997). Alpha-glucosidase inhibitors. *Endocrinology and Metabolism Clinics of North America*.

[46] Tadera K., Minami Y., Takamatsu K., Matsuoka T. (2006). Inhibition of *α*-glucosidase and *α*-amylase by flavonoids. *Journal of Nutritional Science and Vitaminology*.

[47] Lee W. K., Wong L. L., Loo Y. Y., Kasapis S., Huang D. J. (2010). Evaluation of different teas against starch digestibility by mammalian glycosidases. *Journal of Agricultural and Food Chemistry*.

[48] Liu Q., Yang Q.-M., Hu H.-J. (2014). Bioactive diterpenoids and flavonoids from the aerial parts of *Scoparia dulcis*. *Journal of Natural Products*.

[49] Mishra M. R., Behera R. K., Jha S. (2011). A brief review on phytoconstituents and ethnopharmacology of *Scoparia dulcis* Linn. (Scrophulariaceae). *International Journal of Phytomedicine*.

[50] Krey G., Braissant O., L'Horset F. (1997). Fatty acids, eicosanoids, and hypolipidemic agents identified as ligands of peroxisome proliferator-activated receptors by coactivator-dependent receptor ligand assay. *Molecular Endocrinology*.

[51] Kuroda M., Mimaki Y., Honda S., Tanaka H., Yokota S., Mae T. (2010). Phenolics from *Glycyrrhiza glabra* roots and their PPAR-*γ* ligand-binding activity. *Bioorganic and Medicinal Chemistry*.

[52] Quang T. H., Ngan N. T. T., Minh C. V. (2011). Effect of triterpenes and triterpene saponins from the stem bark of *Kalopanax pictus* on the transactivational activities of three PPAR subtypes. *Carbohydrate Research*.

[53] Thomas M. C., Jandeleit-Dahm K. A., Tikellis C. (2012). The renoprotective actions of peroxisome proliferator-activated receptors agonists in diabetes. *PPAR Research*.

[54] Asian Association for the Study of Diabetes (2013). Promoting research for better diabetes care in Asia: Kyoto declaration on diabetes. *Journal of Diabetes Investigation*.

[55] Latha M., Pari L., Ramkumar K. M. (2009). Anti-diabetic effects of scoparic acid D isolated from *Scoparia dulcis* in rats with streptozotocin-induced diabetes. *Natural Product Research*.

[56] Sharma K. R., Adhikari A., Hafizur R. M. (2015). Potent insulin secretagogue from *Scoparia dulcis* linn. of Nepalese origin. *Phytotherapy Research*.

[57] Brownlee M. (2001). Biochemistry and molecular cell biology of diabetic complications. *Nature*.

[58] Brownlee M. (2005). The pathobiology of diabetic complications: a unifying mechanism. *Diabetes*.

[59] Babincová M., Sourivong P. (2001). Free radical scavenging activity of *Scoparia dulcis* extract. *Journal of Medicinal Food*.

[60] Coulibaly A. Y., Kiendrebeogo M., Kehoe P. G. (2011). Antioxidant and anti-inflammatory effects of *Scoparia dulcis* L.. *Journal of Medicinal Food*.

[61] Patra P. K., Debata J., Sravanthi Reddy E., Samal H. B. (2014). Antioxidant study of different extracts of Scoparia dulcis. *International Journal of Pharmacy and Pharmaceutical Sciences*.

[62] Latha M., Pari L. (2003). Modulatory effect of *Scoparia dulcis* in oxidative stress-induced lipid peroxidation in Streptozotocin diabetic rats. *Journal of Medicinal Food*.

[63] Latha M., Pari L., Sitasawad S., Bhonde R. (2004). *Scoparia dulcis*, a traditional antidiabetic plant, protects against streptozotocin induced oxidative stress and apoptosis *in vitro* and *in vivo*. *Journal of Biochemical and Molecular Toxicology*.

[64] Pari L., Latha M. (2004). Protective role of *Scoparia dulcis* plant extract on brain antioxidant status and lipidperoxidation in STZ diabetic male Wistar rats. *BMC Complementary and Alternative Medicine*.

[65] Evans D. A., Hirsch J. B., Dushenkov S. (2006). Phenolics, inflammation and nutrigenomics. *Journal of the Science of Food and Agriculture*.

[66] de Farias Freire S. M., da Silva Emim J. A., Lapa A. J., Souccar C., Brandao Torres L. M. (1993). Analgesic and antiinflammatory properties of *Scoparia dulcis* L. extracts and glutinol in rodents. *Phytotherapy Research*.

[67] de Medeiros P. M., Ladio A. H., Albuquerque U. P. (2013). Patterns of medicinal plant use by inhabitants of Brazilian urban and rural areas: a macroscale investigation based on available literature. *Journal of Ethnopharmacology*.

[68] Paulino R. D. C., Henriques G. P. D. S. A., Moura O. N. S., Coelho M. D. F. B., Azevedo R. A. B. (2011). Medicinal plants at the Sítio do Gois, Apodi, Rio Grande do Norte State, Brazil. *Brazilian Journal of Pharmacognosy*.

[69] Michel J., Duarte R. E., Bolton J. L. (2007). Medical potential of plants used by the Q'eqchi Maya of Livingston, Guatemala for the treatment of women's health complaints. *Journal of Ethnopharmacology*.

[70] Souza R. K. D., da Silva M. A. P., de Menezes I. R. A., Ribeiro D. A., Bezerra L. R., Souza M. M. D. A. (2014). Ethnopharmacology of medicinal plants of carrasco, northeastern Brazil. *Journal of Ethnopharmacology*.

[71] Sala E., Guasch L., Iwaszkiewicz J. (2011). Identification of human IKK-2 inhibitors of natural origin (Part II): *In Silico* prediction of IKK-2 inhibitors in natural extracts with known anti-inflammatory activity. *European Journal of Medicinal Chemistry*.

[72] Israël A. (2010). The IKK complex, a central regulator of NF-*κ*B activation. *Cold Spring Harbor Perspectives in Biology*.

[73] Zulfiker A. H. M., Mahbubur Rahman M. M., Hossain M. K., Hamid K., Mazumder M. E. H., Rana M. S. (2010). *In vivo* analgesic activity of ethanolic extracts of two medicinal plants—*Scoparia dulcis* L. and *Ficus racemosa* Linn. *Biology and Medicine*.

[74] Ratnasooriya W. D., Galhena G., Liyanage S. S. P., Jayakody J. R. A. C., Ediriweera E. R. H. S. S. (2003). Analgesic and antihyperalgesic effects of *Scoparia dulcis* decoction in rats. *Journal of Tropical Medicinal Plants*.

[75] Bourdy G., Oporto P., Gimenez A., Deharo E. (2004). A search for natural bioactive compounds in Bolivia through a multidisciplinary approach: part VI. Evaluation of the antimalarial activity of plants used by Isoceño-Guaraní Indians. *Journal of Ethnopharmacology*.

[76] Moniruzzaman M., Atikur Rahman M., Ferdous A. (2015). Evaluation of sedative and hypnotic activity of ethanolic extract of *Scoparia dulcis* Linn.. *Evidence-Based Complementary and Alternative Medicine*.

[77] Babincová M., Schronerová K., Sourivong P. (2008). Antiulcer activity of water extract of *Scoparia dulcis*. *Fitoterapia*.

[78] Abere T. A., Okoye C. J., Agoreyo F. O. (2015). Antisickling and toxicological evaluation of the leaves of *Scoparia dulcis* Linn (Scrophulariaceae). *BMC Complementary and Alternative Medicine*.

[79] Serjent G. R. (2001). *Sickle Cell Disease*.

[80] Imaga N. A. (2013). Phytomedicines and nutraceuticals: alternative therapeutics for sickle cell anemia. *The Scientific World Journal*.

[81] Abere T. A., Egharevba C. O., Chukwurah I. O. (2014). Pharmacognostic evaluation and antisickling activity of the leaves of *Securinega virosa* Roxb. ex Willd (Euphorbiaceae). *African Journal of Biotechnology*.

[82] Coulibaly A. Y., Hashim R., Sulaiman S. F., Sulaiman O., Ang L. Z. P. (2014). Chemical composition and antimicrobial potential of selected medicinal plants. *International Journal of Pharma and Bio Sciences*.

[83] Niveditha R., Prabavathy D. (2015). Effect of ethanolic extract of *Scoparia dulcis* leaves on the virulence factors of uropathogenic *Escherichia coli* and *Staphylococcus aureus*. *Der Pharmacia Lettre*.

[84] Uma G., Najila Banu A., Sathica Taj J., Josephine Benedit Bai U. (2014). Phytochemical screening and antibacterial activity of *Scoparia dulcis* extracts. *Asian Journal of Pharmaceutical and Clinical Research*.

[85] Zulfiker A. H. Md., Siddiqua M., Nahar L. (2011). *In vitro* antibacterial, antifungal and cytotoxic activity of *Scoparia dulcis* L.. *International Journal of Pharmacy and Pharmaceutical Sciences*.

