# Corrigendum to “Antidiabetic Properties, Bioactive Constituents, and Other Therapeutic Effects of* Scoparia dulcis*”

**DOI:** 10.1155/2017/2535014

**Published:** 2017-07-20

**Authors:** Evidence-Based Complementary and Alternative Medicine

The article titled “Antidiabetic Properties, Bioactive Constituents, and Other Therapeutic Effects of* Scoparia dulcis*” [1] was found to contain some material from the following published articles:

- Paragraph 1: Reuters 2016 [2], uncited and not quoted
- Paragraph 2: Li et al., Journal of Ethnopharmacology 2004 [3], uncited and not quoted
- Section 3.2.1: Lee et al., Journal of Agricultural and Food Chemistry 2010 [4], and Liu et al., Journal of Agricultural and Food Chemistry 2011 [5], uncited and not quoted
- Section 3.2.3: references 55 and 56, cited but not quoted
- Section 4.2: attributed to reference 65 not reference 71, not quoted
- Section 4.3: reference 76, cited but not quoted
- Section 5.6: attributed to references 79–81 not reference 78, not quoted

The authors Geethi Pamunuwa and D. Nedra Karunaratne say they were unaware of the reuse and agree with publishing this corrigendum; the corresponding author Viduranga Waisundara does not agree.
